# Leukocyte Adhesion Molecules in Diabetic Retinopathy

**DOI:** 10.1155/2012/279037

**Published:** 2011-11-02

**Authors:** Kousuke Noda, Shintaro Nakao, Susumu Ishida, Tatsuro Ishibashi

**Affiliations:** ^1^Laboratory of Ocular Cell Biology and Visual Science, Hokkaido University Graduate School of Medicine, N-15, W-7, Kita-ku, Sapporo 060-8638, Japan; ^2^Department of Ophthalmology, Hokkaido University Graduate School of Medicine, N-15, W-7, Kita-ku, Sapporo 060-8638, Japan; ^3^Department of Ophthalmology, Graduate School of Medical Sciences, Kyushu University, Fukuoka 812-8582, Japan

## Abstract

Diabetes is a systemic disease that causes a number of metabolic and physiologic abnormalities. One of the major microvascular complications of diabetes is diabetic retinopathy (DR), a leading cause of blindness in people over age 50. The mechanisms underlying the development of DR are not fully understood; however, extensive studies have recently implicated chronic, low-grade inflammation in the pathophysiology of DR. During inflammation leukocytes undergo sequential adhesive interactions with endothelial cells to migrate into the inflamed tissues, a process known as the “leukocyte recruitment cascade” which is orchestrated by precise adhesion molecule expression on the cell surface of leukocytes and the endothelium. This paper summarizes the recent clinical and preclinical works on the roles of leukocyte adhesion molecules in DR.

## 1. Introduction

Inflammation is a nonspecific, defensive response of the body to tissue injury in which leukocytes are recruited to inflamed tissues. In acute inflammation, leukocytes clear away invading agents and degrade necrotized tissue components, generally contributing to tissue repair. However, if inflammation persists chronically, leukocytes can damage tissues by prolonged secretion of chemical mediators and toxic oxygen radicals. There is an accumulating body of evidence showing that leukocytes play a significant role in the pathogenesis of a number of vision-threatening retinal diseases, such as glaucoma, age-related macular degeneration, and diabetic retinopathy (DR) [1]. This paper will present important findings from the growing amount of research on inflammation specifically in relation to DR.

DR is one of the main microvascular complications of diabetes and one of the most common causes of blindness in people over age 50. Recent studies have elucidated that chronic, low-grade inflammation underlies much of the vascular complications of DR [2, 3]. Microscopic inflammatory responses, such as vessel dilation, vascular leakage, and leukocyte recruitment, occur in the diabetic retina and are implicated in the development of DR [4]. 

For instance, leukocyte adhesion molecules are upregulated in the vessels of the diabetic retina and choroid, and consequently inflammatory cells accumulate in the chorioretinal tissues [5]. Indeed, extensive accumulation of polymorphonuclear leukocytes has been observed in the lumen of microaneurysms, a cause of retinal vascular leakage, in human type 1 diabetic subjects [6, 7]. Correlations between elevated numbers of accumulated leukocytes and capillary damage have been shown in postmortem DR patients [6] and in spontaneously diabetic monkeys [8]. Preclinical studies using animal models of early DR have also revealed that leukocytes adhering to the endothelium damage endothelial cells and increase the vascular permeability of retinal vessels [9, 10]. Leukocytes have also been shown to be present in fibrovascular membranes, a characteristic feature of the pathologic changes associated with proliferative diabetic retinopathy (PDR) [11]. It has been furthermore reported that T lymphocytes infiltrate the fibrovascular membrane [12] and that this infiltration correlates well with the severity of retinopathy and poor visual prognosis [13]. Taken together, these lines of evidence indicate that leukocytes disrupt the homeostasis of the vasculature and facilitate proliferative damage in DR. 

The following sections describe the leukocyte recruitment cascade, which is regulated by a series of adhesion molecules, and present the emerging findings regarding the adhesion molecules specifically involved in DR.

## 2. Leukocyte Recruitment Cascade

**Figure 1:**
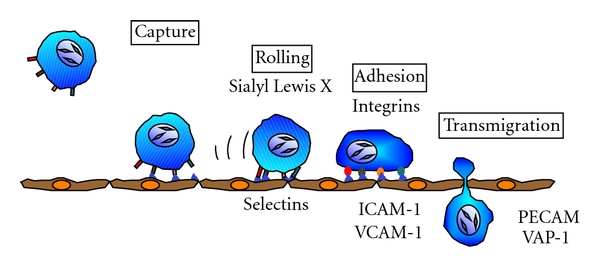
Leukocyte recruitment to the vessel wall.

The recruitment of leukocytes from circulating blood into tissues is crucial for the inflammatory response. During the process, a number of well-studied adhesion molecules on the endothelium sequentially interact with their ligands expressed on the cell surface of leukocytes. The interaction between adhesion molecules and ligands occurs in a cascade-like fashion, guiding leukocytes from the circulation to the extravascular space, that is, through the steps of leukocyte rolling, firm adhesion, and transmigration (Figure 1). 

The selectin family of adhesion molecules mediates the capture and rolling steps of leukocytes along the endothelial cells. The selectins consist of three members of C-type lectins: P-, E-, and L-selectin [14]. P-selectin, stored in the granules of endothelial cells and platelets, translocates rapidly to the cell surface in response to several inflammatory stimuli. Whereas all of the selectins bind to sialyl-Lewis X carbohydrate ligands, such as P-selectin glycoprotein ligand-1 (PSGL-1), interaction between P-selectin and PSGL-1 is responsible for a major part of the leukocyte rolling in inflammation [15]. E-selectin, a heavily glycosylated transmembrane protein, is present exclusively in endothelial cells and is increased by stimulation of representative inflammatory cytokines, such as tumor necrosis factor- (TNF-) *α* and interleukin- (IL-) 1*β* [16]. These inflammatory cytokines are also reported to induce the expression of P-selectin on the endothelium [17, 18]. L-selectin is expressed on many subclasses of leukocytes [14] and binds to endothelial ligands containing sulfated sialyl Lewis X [19].

After the selectins have initiated leukocyte rolling along the surface of the endothelium, a different set of adhesion molecules comes into play to reduce the leukocyte-rolling velocity and allow the leukocytes to firmly adhere to the endothelial surface. This firm adhesion step is largely mediated by molecules of the immunoglobulin superfamily, such as intercellular adhesion molecule- (ICAM-) 1 and vascular cell adhesion molecule- (VCAM-) 1, expressed on endothelial cells and by those of the integrin family expressed constitutively on leukocytes as well as on many other types of cells. The principal integrins that bind to endothelial ICAM-1 are LFA-1 (CD11a/CD18) and Mac-1 (CD11b/CD18), while VLA-4 (CD49d/CD29) is the integrin that binds to endothelial VCAM-1 [20]. Recent studies have revealed that the binding of selectins to PSGL-1 increase the affinity of LFA1 to ICAM-1, indicating that rolling step facilitates the next events, leukocyte adhesion to the endothelial surface [21].

Upon achievement of stable adhesion to the endothelial surface, the leukocytes extravasate between endothelial cells along the intercellular junctions. Platelet endothelial cell adhesion molecule- (PECAM-)1 is an immunoglobulin superfamily member expressed at endothelial cell-cell junctions that mediates this leukocyte transmigration, particularly that of monocytes and neutrophils. In addition, vascular adhesion protein- (VAP-) 1, a 170 kDa homodimeric sialylated glycoprotein, is an endothelial adhesion molecule regulating the transmigration step of lymphocytes, monocytes, and polymorphonuclear leukocytes. VAP-1 was originally discovered in inflamed synovial vessels [22], and; thereafter, it was revealed that it is also expressed on the vascular endothelial cells in tissues such as skin, brain, lung, liver, and heart [23–26]. In ocular tissues, VAP-1 was detected on the endothelial cells of retinal and choroidal vessels [27–29].

## 3. Leukocyte Adhesion Molecules in Diabetic Retinopathy

Clinically, DR is divided into two stages based on the proliferative status of the retinal vasculature: the non-proliferative stage (NPDR) and the proliferative stage (PDR). In NPDR, the early stage of DR, the development of retinopathy begins with vascular lesions that involve pericyte loss, basement membrane thickening, capillary microaneurysms, and obliteration of capillaries [11]. The obliterated capillaries reduce the amount of retinal perfusion and, therefore, lead to ischemic changes in the diabetic retina. This ischemia causes neovascularization in the retina and/or the optic disk. PDR, the later stage of DR, is characterized by retinal neovascularization associated with the formation of a fibrovascular membrane at the vitreoretinal interface. Tractional change against the fibrovascular membrane leads to further severe complications, such as vitreous hemorrhage and tractional and/or rhegmatogenous retinal detachments. Clinical and preclinical studies have provided evidence that leukocyte adhesion molecules play a significant role during both stages of DR.

## 4. Selectins

The data regarding the role of the selectin family specifically in the retinal vessels in DR turns out to be rather intriguing, especially in light of the selectin family's generally known responsibility for leukocyte rolling as described above. Reports show, for instance, that P-selectin is upregulated in the choroidal vessels of diabetic patients, but it is not upregulated in their retinal vessels [5]. Moreover, E-selectin has not been detected in the chorioretinal tissues of either diabetic or nondiabetic patients [5]. On the leukocyte side, a significant decrease in the amount of surface L-selectin expression has been observed in patients with diabetic microangiopathy in comparison with diabetic patients without microangiopathic complications and healthy controls [30]. 

The reason for this pattern of diminished selectin expression on the leukocytes and endothelial cells in DR might be due to the specific nature of the inflammation in DR, that is, in its chronic, low-grade nature. It is known, for instance, that selectins are removed from the cell surface of leukocytes and endothelia through proteolytic shedding during inflammation [31], and this seems to be what is occurring under the longstanding and subclinical inflammatory conditions of DR. Indeed, elevated serum levels of soluble adhesion molecules have been reported in patients with DR; the serum level of soluble E-selectin, for example, is reportedly increased in patients with diabetes [32, 33] and correlates with the progression of DR [34, 35]. It has also been demonstrated that patients with PDR show higher serum levels of soluble P-selectin [36]. 

As for the mechanisms, it has been reported that a disintegrin and metalloproteinase (ADAM) 8, one of the major ectodomain shedding proteinases, is upregulated dur-ing pathological neovascularization, and its overexpression facilitates the shedding of E-selectin [37]. Similarly, during inflammation, L-selectin is shed by ADAM17, which is upregulated in response to inflammatory stimulation [38, 39]. 

In the vitreous, the level of soluble E-selectin was considerably higher in subjects with PDR [40]. Furthermore, vitreous levels of soluble E-selectin in eyes with PDR complicated by traction retinal detachment were significantly increased in comparison with the eyes with vitreous hemorrhage alone [41]. Interestingly, it has been shown that soluble E-selectin stimulates retinal capillary endothelial cell migration [42] and promotes angiogenesis through a sialyl-Lewis-X-dependent mechanism [43].

Accordingly, these various lines of evidence indicate that the shedding of selectins is enhanced on the endothelium during the progression of diabetes and that the soluble form of selectin proteins has the potential to be a clinically useful biomarker of the severity of DR; E-selectin, in particular, may also serve as a proangiogenic factor.

## 5. ICAM-1

While the selectin expression on leukocytes and endothelial cells appears low in DR, other molecules may be compensating for the adhesion gaps. Several studies, for instance, suggest that ICAM-1 and its binding partners are operative in DR and may serve as potential targets for therapeutic interventions. Indeed, ICAM-1 is found to be highly expressed in the blood vessels of the retina, choroid and fibrovascular membrane in patients with diabetes [5, 44], and its expression correlates with the number of migrated neutrophils in the retina and choroid of these patients [5], indicating that elevated ICAM-1 facilitates leukocyte recruitment and the vascular complications in DR. In accord with these clinical observations, ICAM-1 is increased in the retinal vessels in an animal model of DR, and blockade of ICAM-1 attenuated leukostasis, endothelial cell death, and vascular leakage in the retinal vessels of the diabetic animals [10]. 

Not only ICAM-1 but also LFA-1 (CD11a/CD18) and Mac-1 (CD11b/CD18) ligands for ICAM-1 are upregulated in patients with diabetes. The *β*-integrin subunit CD18 is increased in patients with DR [45], and likewise significant increases in *α*-integrin subunits CD11a [46] and CD11b [30] are found in these patients. Consequently, these data indicate that ICAM-1 and its ligands are important and interruption of either component of the integrin-ICAM-1 interaction may be beneficial in preventing the deterioration of DR. However, it was also reported that the effect of ICAM-1 depletion was limited to prevent angiogenesis in oxygen-induced retinopathy model [47]. Further investigation is still required to elucidate the role of integrin-ICAM-1 interaction in DR.

## 6. VCAM-1

Similar to ICAM-1, a role for endothelial VCAM-1 in DR is also emerging, although there has not been as much research conducted on VCAM-1 as with ICAM-1. Before its potential role in DR was examined, it first came to light that VCAM-1 had been involved in the macrovascular complications of diabetes [48]; however, it has since been revealed that the interaction of VCAM-1 with its ligand, integrin VLA-4, is important in the development of DR. For instance, it has been demonstrated in an animal model of DR that hyperglycemia upregulates VCAM-1 expression in the retinal vessels [49]. Also, in an animal model of early DR, it has been found that VLA-4-mediated leukocyte adhesion to the retinal vessels is significantly increased, and blockade of VLA-4 attenuates vascular leakage and production of inflammatory cytokines [50].

In addition, increased serum levels of soluble VCAM-1 have been found in type 2 diabetic patients with microvascular complications, similar to E-selectin [32], and levels of soluble VCAM-1 are elevated in the vitreous of DR patients as well [40, 41]. Notably, it has been shown that soluble VCAM-1 acts on endothelial cells as an angiogenic factor through a VLA-4-dependent mechanism, in common with E-selectin [42, 43], suggesting that blockade of both soluble adhesion molecules, soluble forms of E-selectin and VCAM-1, could have a beneficial effect on DR.

## 7. VAP-1

Along with ICAM-1, VAP-1 seems also to be a key player in the inflammation associated with DR, as demonstrated through several lines of evidence. Blockade of VAP-1, for example, significantly reduces the transmigration and/or capillary entrapment of leukocytes in the retina in an animal model of DR [51]. Transmigrated leukocytes under pathological conditions are thought to play an important role in neovascularization through secretion of VEGF [52]. Furthermore, leukocytes firmly adhering to capillary endothelial cells induce apoptotic changes in the endothelial cells [53]. Therefore, VAP-1 seems to be locally involved in the pathogenesis of DR by mediating leukocyte recruitment.

In further support of this conclusion, VAP-1 also exists as a soluble form in plasma, and much attention has recently been paid to the elevated serum concentration of soluble VAP-1 in patients with diabetes [54, 55]. Interestingly, besides its role as an adhesion molecule, VAP-1 has also an enzymatic function as a semicarbazide-sensitive amine oxidase (SSAO), which converts aliphatic primary monoamines to the corresponding aldehydes with the release of hydrogen peroxide and ammonia [56]. Metabolites generated by VAP-1/SSAO, for example, hydrogen peroxide and methylglyoxal from aminoacetone are known to be involved in cellular oxidative stress and advanced glycation end-product formation, both of which are crucial for the pathogenesis of diabetic retinopathy [57, 58]. Therefore, it seems likely that soluble VAP-1 in the serum and vitreous may promote vascular complications in DR. In fact, patients with DR display significantly higher plasma VAP-1/SSAO activities compared to patients without DR [55]. Further investigation using human ocular samples may aid in a better understanding of the role of VAP-1 in DR.

## 8. Conclusions

The pathogenesis of DR is not entirely known. However, based on the preceding discussion, growing evidence supports a role for leukocytes and their adhesion molecules in the development of DR. In addition, soluble adhesion molecules may contribute to DR by acting as angiogenic factors or enzymes. Specific inhibition of leukocyte adhesion molecules could be of therapeutic value for diabetic patients.
